# Intrinsic fluorescence of the clinically approved multikinase inhibitor nintedanib reveals lysosomal sequestration as resistance mechanism in FGFR-driven lung cancer

**DOI:** 10.1186/s13046-017-0592-3

**Published:** 2017-09-07

**Authors:** Bernhard Englinger, Sebastian Kallus, Julia Senkiv, Daniela Heilos, Lisa Gabler, Sushilla van Schoonhoven, Alessio Terenzi, Patrick Moser, Christine Pirker, Gerald Timelthaler, Walter Jäger, Christian R. Kowol, Petra Heffeter, Michael Grusch, Walter Berger

**Affiliations:** 1 Institute of Cancer Research and Comprehensive Cancer Center, Department of Medicine I, Medical University of Vienna, Borschkegasse 8a, A-1090 Vienna, Austria; 2 Institute of Inorganic Chemistry, University of Vienna, Waehringer Str. 42, A-1090 Vienna, Austria; 3 Research Cluster “Translational Cancer Therapy Research”, University of Vienna, Waehringer Strasse 42, A-1090 Vienna, Austria; 4 Institute of Cell Biology NAS of Ukraine, Drahomanova str 14/16, 79005 Lviv, Ukraine; 5 Department of Pharmacology and Toxicology, University of Vienna, Althanstr. 14, 1090 Vienna, Austria; 6 Department of Pharmaceutical Chemistry, Division of Clinical Pharmacy and Diagnostics, University of Vienna, Althanstrasse 14, A-1090 Vienna, Austria

**Keywords:** FGFR1, Nintedanib, Fluorescence, Lysosomes, Resistance

## Abstract

**Background:**

Studying the intracellular distribution of pharmacological agents, including anticancer compounds, is of central importance in biomedical research. It constitutes a prerequisite for a better understanding of the molecular mechanisms underlying drug action and resistance development. Hyperactivated fibroblast growth factor receptors (FGFRs) constitute a promising therapy target in several types of malignancies including lung cancer. The clinically approved small-molecule FGFR inhibitor nintedanib exerts strong cytotoxicity in FGFR-driven lung cancer cells. However, subcellular pharmacokinetics of this compound and its impact on therapeutic efficacy remain obscure.

**Methods:**

3-dimensional fluorescence spectroscopy was conducted to asses cell-free nintedanib fluorescence properties. MTT assay was used to determine the impact of the lysosome-targeting agents bafilomycin A1 and chloroquine combined with nintedanib on lung cancer cell viability. Flow cytometry and live cell as well as confocal microscopy were performed to analyze uptake kinetics as well as subcellular distribution of nintedanib. Western blot was conducted to investigate protein expression. Cryosections of subcutaneous tumor allografts were generated to detect intratumoral nintedanib in mice after oral drug administration.

**Results:**

Here, we report for the first time drug-intrinsic fluorescence properties of nintedanib in living and fixed cancer cells as well as in cryosections derived from allograft tumors of orally treated mice. Using this feature in conjunction with flow cytometry and confocal microscopy allowed to determine cellular drug accumulation levels, impact of the ABCB1 efflux pump and to uncover nintedanib trapping into lysosomes. Lysosomal sequestration - resulting in an organelle-specific and pH-dependent nintedanib fluorescence - was identified as an intrinsic resistance mechanism in FGFR-driven lung cancer cells. Accordingly, combination of nintedanib with agents compromising lysosomal acidification (bafilomycin A1, chloroquine) exerted distinctly synergistic growth inhibitory effects.

**Conclusion:**

Our findings provide a powerful tool to dissect molecular factors impacting organismal and intracellular pharmacokinetics of nintedanib. Regarding clinical application, prevention of lysosomal trapping via lysosome-alkalization might represent a promising strategy to circumvent cancer cell-intrinsic nintedanib resistance.

**Electronic supplementary material:**

The online version of this article (10.1186/s13046-017-0592-3) contains supplementary material, which is available to authorized users.

## Background

Fibroblast growth factor receptors (FGFRs) are a family of receptor tyrosine kinases (RTKs) that play an important role in a plethora of biological functions including tissue development and homeostasis [1, 2]. Activation of the FGF/FGFR signaling circuitry employs multiple downstream signaling modules that regulate gene expression in order to promote cell survival, proliferation, migration as well as differentiation [3, 4]. In various tissue types, somatic gain-of-function alterations of FGFRs have been described to promote cancer development [5–8]. These alterations include genetic amplification, activating mutations as well as oncogenic fusion genes and are present with varying frequencies in carcinomas of the lung, breast and ovary, in gastric and urothelial cancer as well as in glioblastoma, rhabdomyosarcoma and Ewing sarcoma [9–12].

These findings have incentivized the development of therapeutic agents targeting oncogenic FGFR signaling [11, 13]. Nintedanib (Vargatef®) is a small-molecule tyrosine kinase inhibitor (TKI) targeting FGFR, vascular endothelial growth factor receptor (VEGFR) as well as platelet-derived growth factor receptor (PDGFR). Nintedanib is clinically approved for second-line therapy of non-small cell lung cancer (NSCLC) of adenocarcinoma histology in Europe and several other FGFR-targeting drugs are currently being evaluated for their clinical efficacy in various other tumor types ([14–16], www.clinicaltrials.gov). The molecular pharmacology and tissue distribution of several FGFR inhibitors is well established [17–20]. In contrast, dynamics of intracellular distribution remain obscure, largely accounted for by lack or extreme complexity of technologies with resolutions high enough to be capable of detecting these agents at subcellular and cell organelle level [21–24].

Nintedanib is a lipophilic compound and, consequently, believed to be able to diffuse freely in its neutral form through lipid-bilayer membranes [14]. In addition, it exhibits weakly basic properties (pK = 7.9 [25]) and can, thus, be fully protonated in low pH environments. Lysosomes are highly acidic organelles present in virtually all mammalian cell types. These organelles exhibit multiple functions including the regulation of homeostasis of intracellular metabolites through the process of autophagy and breakdown of macromolecules [26, 27]. Regarding signaling of RTKs like FGFR or epidermal growth factor receptor (EGFR), lysosomes are central to the breakdown of the internalized growth factor/RTK complexes and recycling of the RTK molecules back to the plasma membrane [2, 28, 29]. Another important lysosomal function is passive protonation-based trapping of hydrophobic xenobiotics, including chemotherapeutics, sequestering these agents from their target sites and thus decreasing their cytotoxic effects [30–32].

Besides membrane permeability of pharmacological agents, their sequestration to subcellular compartments is a major determinant of target interaction efficacy and, consequently, resistance [32]. In this study, we describe the fluorescence-based, label-free and organelle-specific intracellular visualization and quantification of the clinically approved multikinase TKI nintedanib in lung cancer cells in vitro as well as in tumor allografts of orally treated mice. Based on this unique characteristic, nintedanib transport by ABCB1 was exemplarily dissected and inactivation by lysosomal trapping was identified to represent a novel resistance mechanism limiting nintedanib activity in FGFR1 amplification-driven lung cancer.

## Methods

### Cell culture

The human lung cancer cell lines NCI-H1703, NCI-H520 (non-small cell lung cancer, NSCLC) and DMS114 (small cell lung cancer, SCLC) were purchased from American Type Culture Collection (Manassas, VA, USA) and grown in RPMI-1640 supplemented with 10% fetal calf serum (FCS, PAA, Linz, Austria) at 37 °C and 5% CO_2_. Cells were authenticated by array comparative genomic hybridization (aCGH) and checked for *Mycoplasma* contamination (Mycoplasma Stain kit, Sigma, St. Louis, Missouri, USA) on a regular basis.

### Drugs and chemicals

Nintedanib, elacridar and chloroquine were purchased from Selleckchem (Munich, Germany). LysoTracker® Red was obtained from Thermo Fisher Scientific (Waltham, MA, USA), bafilomycin A1 was purchased from Sigma.

### Fluorescence spectroscopy

Three dimensional-fluorescence spectra were recorded on a Horiba FluoroMax®-4 spectrofluorometer (Kyoto, Japan) and processed using the FluorEssence v3.5 software package. Stock solutions of nintedanib-ethanesulfonate in dimethylsulfoxide (DMSO) were diluted with phosphate-buffered saline (PBS) (10 mM, pH 7.4) to 15 μM (final DMSO concentration 1%) and the fluorescence spectra were measured at excitation wavelengths from 220 nm to 420 nm while the emission was within the range of 240–700 nm. Scans were run at room temperature with excitation and emission slit widths of 5 nm.

### Cell viability assay

To determine cell viability upon inhibition of FGFR1, 3 × 10^3^ cells were seeded in 96-well plates and incubated overnight. Cells were exposed to the indicated concentrations of nintedanib in the presence or absence of the indicated concentrations of elacridar, bafilomycin A1 or chloroquine. After 72 h, cell survival was determined using the 3-(4,5-dimethylthiazol-2-yl)-2,5-diphenyltetrazolium bromide (MTT)-based vitality assay (EZ4U, Biomedica, Vienna, Austria). Dose-response curves were plotted using GraphPad Prism software (La Jolla, CA, USA). IC_50_ values were determined from non-linear regression curve-fitting (sigmoidal dose-response with variable slope) in GraphPad Prism and indicate drug concentrations that resulted in a 50% reduced cell viability in comparison to untreated controls. Drug synergism was determined using Calcu Syn software (Biosoft, Ferguson, MO, USA) according to Chou-Talalay and expressed as combination index (CI) [33]. A CI value of <0.9 was considered a synergistic effect, a CI value between 0.9–1.1 indicates additivity and a CI value greater than 1.1 was considered an antagonistic effect.

### Flow cytometry

5 × 10^5^ cells were resuspended in serum-free RPMI medium containing 2.09 mg/ml 4-morpholine-propanesulfonic acid (MOPS, Sigma) and 15 mM 4-(2-hydroxyethyl)piperazine-1-ethanesulfonic acid (HEPES, Sigma). Following a 1 h preincubation with 10 μM elacridar or 1 μM bafilomycin A1, cells were treated with the indicated concentrations of nintedanib. Intracellular drug accumulation was measured on a LSRFortessa flow cytometer (BD Biosciences, East Rutherford, NJ, USA) at the indicated time-points. Compound fluorescence was detected using 405 nm and 488 nm laser excitation wavelengths, and Horizon V450 (450/40 nm) and FITC (530/30 nm) bandpass emission filters, respectively. Data were analyzed using Flowing Software (University of Turku, Finland) and are depicted as relative increase in fluorescence intensities (arbitrary units, a.u.) compared to untreated controls.

### Live cell microscopy

5 × 10^4^ NCI-H1703 cells were seeded in 8-well chamber slides (Ibidi, Martinsried, Germany). After 24 h, cells were treated with 10 μM nintedanib and intracellular drug accumulation was imaged at the indicated time-points on a live cell microscope (Visitron Systems, Puchheim, Germany) using a 40× oil immersion DIC objective and VisiView® software. LEDs were used for widefield DIC and fluorescence (395/25 nm excitation and 460/50 nm bandpass filter for blue (DAPI) fluorescence and 475/34 nm excitation and 525/50 nm bandpass filter for green (FITC) fluorescence) illumination (Visitron Systems). Images were taken with a sCMOS 4.2MPxl digital camera. Increase in nintedanib fluorescence intensity over time was quantified using ImageJ software. The fluorescence signals of individual cells from at least three independent microscopic images were determined and values are depicted as relative fluorescence increase as compared to the 0 min control. To investigate the impact of lysosomal pH on the intralysosomal accumulation of nintedanib, cells were pretreated with 1 μM bafilomycin A1 and 1 μM LysoTracker® Red prior to exposure to 10 μM nintedanib. Cell-free nintedanib fluorescence properties were determined by imaging of the drug in its crystalline form, afterwards dissolved to 1 mM in DMSO and recrystallized after DMSO evaporation in the DIC, FITC and DAPI channels at 40× magnification.

### Western blot analysis

Total cell protein extracts were separated by sodium dodecyl sulfate- polyacrylamide gel electrophoresis (SDS-PAGE) and blotted onto polyvinylidene difluoride membranes (PVDF, Thermo Fisher Scientific). Anti-ß-actin (AC-15) was purchased from Sigma, anti-ABCB1 (C219) from BioLegend (San Diego, CA, USA). Horseradish peroxidase-conjugated secondary antibodies were obtained from Santa Cruz Biotech (Dallas, TX, USA).

### Confocal fluorescence microscopy

5 × 10^3^ cells were seeded in 8-well spot slides (Thermo Fisher Scientific). The next day, cells were coincubated for 1 h with 10 μM nintedanib and LysoTracker® Red. Alternatively, DMS114 cells were incubated for 1 h with 10 μM nintedanib in the presence or absence of 10 μM of the ABCB1 inhibitor elacridar [34]. Cells were fixed with 4% paraformaldehyde (PFA) for 15 min. Images were acquired on an inverted point scanning confocal microscope with PMTs (LSM700, Zeiss, Jena, Germany) using a 63× Plan-Apochromat 63×/1.4 oil immersion objective with Zen2010® software (Zeiss) using 405 nm (nintedanib blue), 488 nm (nintedanib green) or 555 nm (LysoTracker® Red) solid state laser lines for excitation and 420 nm longpass, 556 nm shortpass and 559 nm longpass emission filters, respectively. Colocalization signals derived from nintedanib and LysoTracker® Red were calculated in ImageJ software using the thresholded Manders‘ Colocalization Coefficient (MCC), which corrects for differences in mean signal intensities of the two channels (a MCC of 0 means no colocalization and 1 means perfect colocalization, see [35]). The mean thresholded MCC was derived from ten to twenty individual cells analyzed from at least three independent microscopic images. Statistical significance of overlapping pixel intensities was calculated by ImageJ software using Costes Colocalization Test [36]. A *p*-value of 0.95 or greater was considered to be statistically significant.

### Imaging of intratumoral nintedanib in allograft cryosections

For detection of nintedanib in subcutaneous tumor allografts, 5 × 10^5^ CT26 mouse colon carcinoma cells were injected subcutaneously into the right flank of four 8 weeks old male BALB/c mice. Animal experiments were authorized by the Ethics committee at the Medical University of Vienna and carried out in accordance with the guidelines for the welfare and use of animals in cancer research, as well as meeting the Federation of Laboratory Animal Science Associations (FELASA) guidelines’ definition of humane endpoints and the Arrive guidelines for animal care and protection [37]. Upon tumor formation, two mice received a single oral dose of 100 mg nintedanib per kg bodyweight dissolved in H_2_O containing 0.5% Klucel and 0.1% Tween80 18 days post-engraftment. Two mice received solvent only. Two hours after administration, mice were sacrificed and tumors were embedded and frozen in OCT medium (Sakura Finetek, Staufen, Germany). 5 μm sections were sliced on a cryomicrotome (Thermo Fisher Scientific), fixed with 4% PFA on Superfrost Plus microscope slides (Thermo Fisher Scientific) and counterstained with 4′,6-diamidine-2′-phenylindole dihydrochloride (DAPI, Sigma). Tumor endothelium was visualized on consecutive sections using an anti-MECA-32 antibody (ab27853, Abcam, Cambridge, United Kingdom). Images of overlapping regions of consecutive cryosections were acquired on a Zeiss LSM700 confocal laser scanning microscope using 40× and 63× oil immersion objectives.

### Quantification of intracellular nintedanib levels by high-performance liquid chromatography (HPLC)

10^6^ cells were plated in 6-well plates. The next day, cells were preincubated with 1 μM bafilomycin A1 for 1 h. Subsequently, 10 μM nintedanib was added to cells for 10 or 60 min. Cells were washed with PBS, trypsinized, washed again twice and lysed by repeated freeze/thaw cycles. Samples were analyzed by HPLC using a Dionex UltiMate 3000 system equipped with an L-7250 injector, an L-7100 pump, an L-7300 column oven (set at 35 °C), a D-7000 interface and an L-7400 UV detector (Thermo Fisher Scientific) set at a wavelength of 287 nm. Separation of nintedanib was carried out at 35 °C using a Hypersil BDS-C18 column (5 μm, 250 × 4.6 mm I.D., Thermo Fisher Scientific), preceded by a Hypersil BDS-C18 precolumn (5 μm, 10 × 4.6 mm I.D.). The mobile phase consisted of a continuous gradient mixed from ion pair buffer, pH 3.0 (50 mM potassium phosphate with phosphoric acid and 5 mM heptane sulfonic acid) (mobile phase A) and acetonitrile (mobile phase B). Mobile phase was filtered through a 0.45 μm filter (HVLP04700, Millipore, Billerica, MA). Mobile phase B linearly increased from 10% acetonitrile (0 min) to 95% at 7 min, at which point it was kept constant until 20 min. The percentage of acetonitrile was decreased within 1 min to 10% to equilibrate the column for 14 min before application of the next sample. Linear calibration curves were performed by spiking drug-free cell culture medium with standard solutions of nintedanib to give a concentration range from 0.05 to 10 μg/ml (average correlation coefficients: >0.999). The limit of quantification (LOQ) for nintedanib was 0.03 μg/ml. Coefficients of accuracy and precision for these compounds were <9%.

### Statistical analysis

Data were analyzed using GraphPad Prism software. If not stated otherwise in the figure legends, one out of at least three independent experiments in triplicate is depicted. So each data point represents the mean ± SD of triplicate values. Statistical evaluation was performed using student’s t-test as well as 1-way or 2-way analysis of variance (ANOVA). Tukey’s post-test was performed to examine differences between drug treatment effects. *P*-values below 0.05 were considered statistically significant. * *p* < 0.05; ** *p* < 0.01; *** *p* < 0.001.

## Results

### The multikinase inhibitor nintedanib exhibits different fluorescence properties under cell-free and cellular conditions

**Fig. 1:**
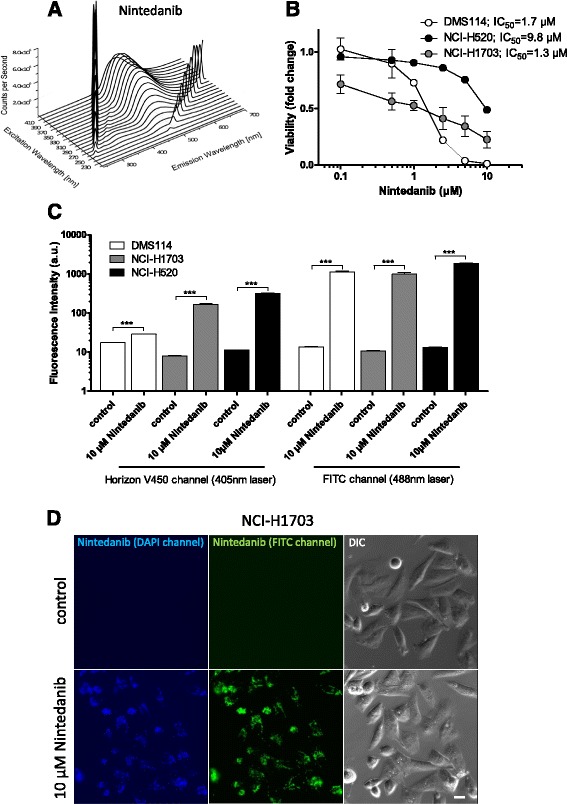
Nintedanib exhibits intracellular and cell-free intrinsic fluorescence activity. **a** Full excitation-emission 3D landscape was obtained by fluorescence spectroscopy to elucidate cell-free fluorescence properties of nintedanib. Spectra are shown for excitation wavelengths from 220 nm to 420 nm. Emission was measured from 240 to 700 nm. 1st and 2nd order Rayleigh scattering can be seen as diagonal ridges. **b** Viability of DMS114, NCI-H520 and NCI-H1703 lung cancer cells was analyzed by MTT assay after 72 h exposure to the indicated concentrations of nintedanib. **c** Intracellular fluorescence activity of 10 μM nintedanib in DMS114, NCI-H1703 and NCI-H520 cells after 1 h exposure was measured by flow cytometry. Fluorescence emission was analyzed using the Horizon V450 channel (450/40 nm bandpass filter) for the 405 nm laser and the FITC channel (530/30 nm bandpass filter) for the 488 nm laser. *** *p* < 0.001, students’s t-test. **d** Blue and green fluorescence activity in NCI-H1703 cells, treated for 1 h with 10 μM nintedanib was analyzed by live cell microscopy. The scale bar indicates 10 μm

With the intention to dissect the intracellular distribution of nintedanib, we investigated whether this multikinase and FGFR-targeting inhibitor might display intrinsic fluorescence activity. Indeed, full excitation-emission 3-dimensional (3D) fluorescence plots revealed a distinct fluorescence pattern of this compound. Under cell-free conditions, nintedanib exhibited an emission maximum at 482 nm when excited at 390 nm (Fig. 1a). Consequently, we investigated whether the observed intrinsic fluorescence activity under cell-free conditions could be utilized to detect this compound label-free in treated cancer cells. To this end, the three FGFR1-driven lung cancer cell lines DMS114, NCI-H520 and NCI-H1703 were used. These cell lines all bear oncogenic amplification of the *FGFR1* gene and are hypersensitive towards FGFR1 inhibitors [38]. Accordingly, also in our analyses IC_50_ values for nintedanib were found in the low-micromolar to nanomolar range (Fig. 1b; 1.97 ± 0.31 μM, 6.67 ± 4.20 μM and 0.98 ± 0.33 μM for DMS114, NCI-H520 and NCI-H1703, respectively). Flow cytometric analysis showed significantly increasing fluorescence signals in the blue portion of the spectrum (450/40 nm bandbass filter) upon nintedanib treatment when excited with the 405 nm laser (Fig. 1c). Interestingly and unexpectedly from cell-free conditions, also 488 nm laser excitation (530/30 nm bandpass filter) of cells treated with nintedanib yielded strong fluorescence activity in the green emission range (Fig. 1c; Additional file 1: Table S1). This effect was also observed in live-cell fluorescence microscopy of treated NCI-H1703 cells (Fig. 1d). This suggests that, based on specific (sub)cellular conditions, the nintedanib molecule or a derivative/state had adopted novel fluorescence properties. Interestingly, fluorescence intensity was distinctly higher when excited with the 488 nm as compared to the 405 nm laser suggesting that either this fluorescence is stronger per se or that an accumulation of the green-fluorescent nintedanib derivative/state had occurred.

### Time- and dose-dependent detection of intracellular nintedanib

**Fig. 2:**
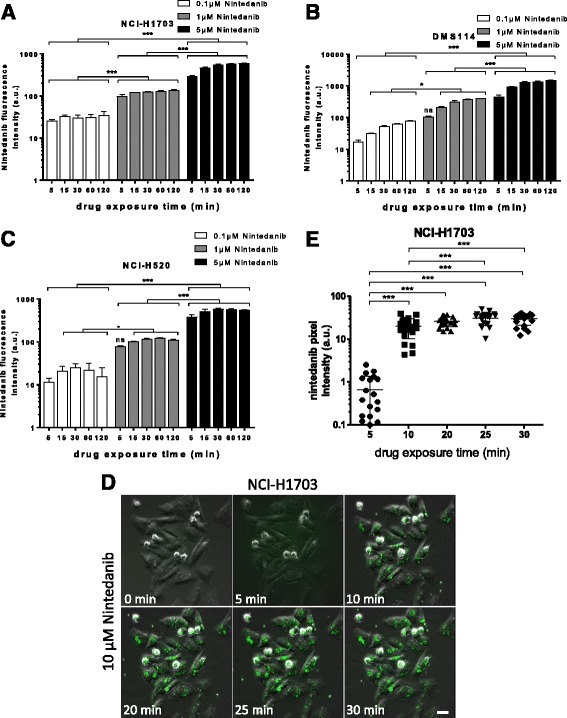
Dose- and time-dependent detection of intracellular nintedanib accumulation by flow cytometry and live cell microscopy. **a**-**c** Time-dependent intracellular fluorescence activity of indicated concentrations of nintedanib in NCI-H1703 (**a**), DMS114 (**b**) and NCI-H520 cells (**c**) was measured by flow cytometry. Nintedanib was detected using the 488 nm laser. Signals are plotted as arbitrary units. Mean autofluorescence values were 6.3, 7.9 and 6.6 for NCI-H1703, DMS114 and NCI-H520, respectively. * *p* < 0.05, *** *p* < 0.001, 2-way ANOVA, Tukey’s post-test. ns, non-significant. Statistical significance is indicated by the asterisks and includes testing of all time-points between each drug concentration. **d** Intracellular accumulation of 10 μM nintedanib in NCI-H1703 cells over time was analyzed by live cell microscopy. The scale bar indicates 10 μm. **e** Quantification of nintedanib pixel intensities of representative micrographs from (D). Signals derived from individual cells are plotted as arbitrary units. The mean value for the 0 min control was 0.003. *** *p* < 0.001, 1-way ANOVA, Tukey’s post-test

The observed fluorescence activity of nintedanib prompted us to investigate whether flow cytometry is a feasible approach to measure intracellular drug accumulation in a dose- and time-dependent manner. Indeed, exposure to nintedanib resulted in a distinct dose- and time-dependent increase in fluorescence intensity in all tested cell lines (Fig. 2a-c). Of note, increasing nintedanib fluorescence intensity over time suggested steady uptake kinetics at least until 2 h after drug administration with the exception of NCI-H520 cells, where the signal peaked after 30 min. We further investigated whether -additionally to flow cytometry- fluorescence microscopy might be another useful approach to monitor the uptake kinetics of the investigated TKI in treated cells. Thus, we performed live cell imaging of NCI-H1703 cells treated with 10 μM nintedanib. Indeed, weak fluorescence was observed already 5 min after drug exposure. At 10 min exposure, distinct fluorescent foci became apparent which increased in intensity in a time-dependent manner (Fig. 2d, e). These data prove that intracellular accumulation of nintedanib and its dose and time-dependent dynamics can be measured by both flow cytometry and fluorescence microscopy. Additionally, the observed foci suggested that the green-fluorescent nintedanib derivative/state accumulated in a yet undefined cell organelle.

### Fluorescence-based detection of reduced intracellular nintedanib levels by ABCB1-mediated efflux

**Fig. 3:**
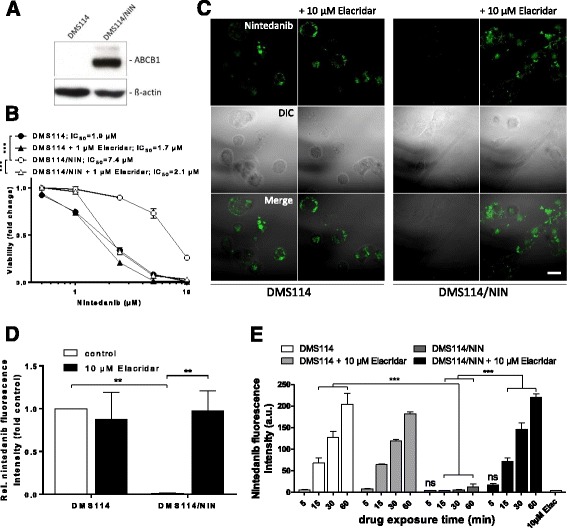
Fluorescence-based monitoring of ABCB1-mediated reduction of intracellular nintedanib levels. **a** Expression of ABCB1 in DMS114 cells and their isogenic nintedanib-resistant counterpart DMS114/NIN was analyzed by Western blot. ß-actin served as loading control. **b** Impact of ABCB1 inhibition by elacridar on the cytotoxic activity of nintedanib in DMS114 cells and their resistant subline was analyzed by MTT assay 72 h after drug exposure. *** *p* < 0.001, 2-way ANOVA, Tukey’s post-test. **c** Impact of 10 μM elacridar on the intracellular accumulation of 10 μM nintedanib in DMS114 and DMS114/NIN cells was analyzed by confocal fluorescence microscopy after 1 h drug exposure. The scale bar indicates 10 μm. **d** Quantification of relative fluorescence intensities of micrographs shown in (**c**) is plotted normalized to nintedanib-treated DMS114 control cells. *** *p* < 0.001, 2-way ANOVA, Tukey’s post-test. **e** Impact of 10 μM elacridar on the intracellular accumulation of 10 μM nintedanib in DMS114 and DMS114/NIN cells was measured at the indicated time-points by flow cytometry using the FITC channel. *** *p* < 0.001, 2-way ANOVA, Tukey’s post-test. *ns* non-significant

We further wanted to verify whether the detected nintedanib fluorescence enables the visualization of decreased intracellular drug accumulation mediated by active drug efflux mechanisms. To this end, we used DMS114 cells and their isogenic subline DMS114/NIN previously selected in our group for acquired nintedanib resistance [39]. This resistance phenotype is mediated by overexpression of the ATP-binding cassette drug efflux transporter B1 (ABCB1, P-glycoprotein), which actively pumps nintedanib out of the cell, thus minimizing its cytotoxic efficacy (Fig. 3a, b). In line, ABCB1 blockade by the allosteric inhibitor elacridar completely restored nintedanib sensitivity (Fig. 3b). In parallel to viability assays, DMS114 and DMS114/NIN cells treated with 10 μM nintedanib for 1 h in the presence or absence of an equimolar concentration of elacridar were also investigated by confocal fluorescence microscopy of PFA-fixed cells. The green fluorescence signal of nintedanib and its spotted pattern were well preserved in fixed cells. In contrast, the fluorescence signal in DMS114/NIN cells was, indeed, virtually absent. However, coincubation with elacridar completely restored nintedanib fluorescence intensity in DMS114/NIN cells while having no significant effects in the parental cell line lacking ABCB1 expression (Fig. 3c, d). In line with this, flow cytometric analysis of cells treated with 10 μM nintedanib confirmed a lack of nintedanib fluorescence in DMS114/NIN cells and accumulation restoration by coincubation with elacridar (Fig. 3e).

### Nintedanib selectively accumulates in lysosomes and is detectable in tumor allografts in vivo

**Fig. 4:**
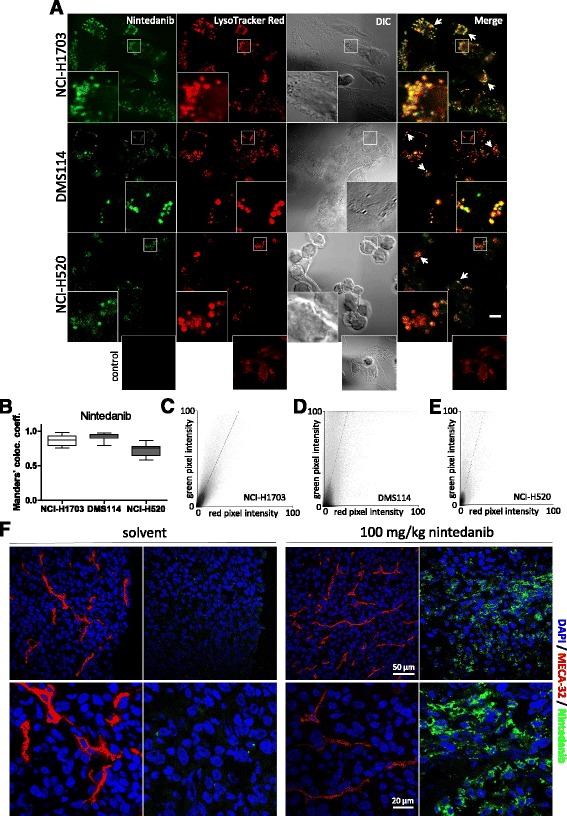
Nintedanib selectively localizes to lysosomes and nintedanib fluorescence is detectable in tumor specimen of treated animals. **a** Subcellular distribution of 10 μM nintedanib in NCI-H1703, DMS114 and NCI-H520 cells after 1 h drug exposure was analyzed by confocal fluorescence microscopy in the FITC channel. LysoTracker® Red served as marker for the lysosomal compartment. Cell boundaries were imaged in the DIC channel. The arrows indicate regions of distinct drug/LysoTracker® Red spatial overlap. The scalebar indicates 10 μm. **b** Colocalization of nintedanib and lysosome-derived signals was determined using thresholded Manders’ Colocalization Coefficient (MCC). **c**-**e** Representative scatter plots showing nintedanib/LysoTracker® Red pixel intensity correlations in NCI-H1703 (**c**), DMS114 (**d**) and NCI-H520 (**e**) cells derived from confocal micrographs shown in Fig. 4a. **f** Intratumoral nintedanib in tissue cryosections detected by confocal fluorescence microscopy using the FITC channel. Mice bearing subcutaneous CT26 tumor allografts received a single oral dose of 100 mg nintedanib per kg bodyweight or solvent. 2 h after drug administration, mice were sacrificed and consecutive cryosections of OCT-embedded tumors were generated. DAPI served as nuclear counterstain. The endothelial marker MECA-32 was stained to visualize tumor microvasculature. Representative micrographs of tumors are shown from the experiment performed in duplicates

The focal appearance of nintedanib fluorescence (compare Fig. 2d) prompted us to further analyze the underlying intracellular drug compartmentalization. The fact that nintedanib is a weakly basic compound which can be fully protonated in a low pH environment led us to hypothesize that preferred sites of intracellular accumulation might be acidic organelles such as lysosomes. Thus, we performed confocal microscopy of nintedanib-treated cells that were simultaneously labelled with LysoTracker® Red (Fig. 4a). Corroborating our hypothesis, spatial overlap of signals originating from nintedanib with LysoTracker® Red was found, suggesting selective nintedanib accumulation in lysosomes. To analyze whether nintedanib signal indeed colocalized with LysoTracker® Red, correlations of overlapping pixel intensities were calculated using thresholded Mander’s colocalization coefficient (MCC). This analysis yielded high correlation coefficients of 0.87, 0.92 and 0.72 for NCI-H1703, DMS114 and NCI-H520, respectively, indicating substantial colocalization between nintedanib- and LysoTracker® Red-derived signals (Fig. 4b). Scatter plots showing drug/LysoTracker® Red pixel intensity correlations for nintedanib are shown in Fig. 4c-e. Additionally, Costes test of overlapping pixel intensities yielded a *p*-value of 1.00, providing statistical evidence for significant colocalization. Thus, we conclude that nintedanib selectively accumulates in lysosomes of treated cells and hence represents a lysosomotropic agent.

To test whether nintedanib is also detectable in tumor tissue in vivo*,* we generated subcutaneous allograft models of the highly tumorigenic mouse colorectal carcinoma cell line CT-26. Indeed, oral treatment resulted in the accumulation of detectable drug levels adjacent to terminal microvasculature of the tumor tissue 2 h post-administration (Fig. 4f). Importantly, the focal appearance of nintedanib in the fixed tumor sections seemed to recapitulate the subcellular, dotted distribution of the drug in cell culture. This illustrates that the intrinsic fluorescence activity might enable sensitive detection of nintedanib not only in vitro*,* but also in vivo in the relevant target tissue following oral administration.

### Inhibition of lysosomal sequestration increases the cytotoxic potential of nintedanib

**Fig. 5:**
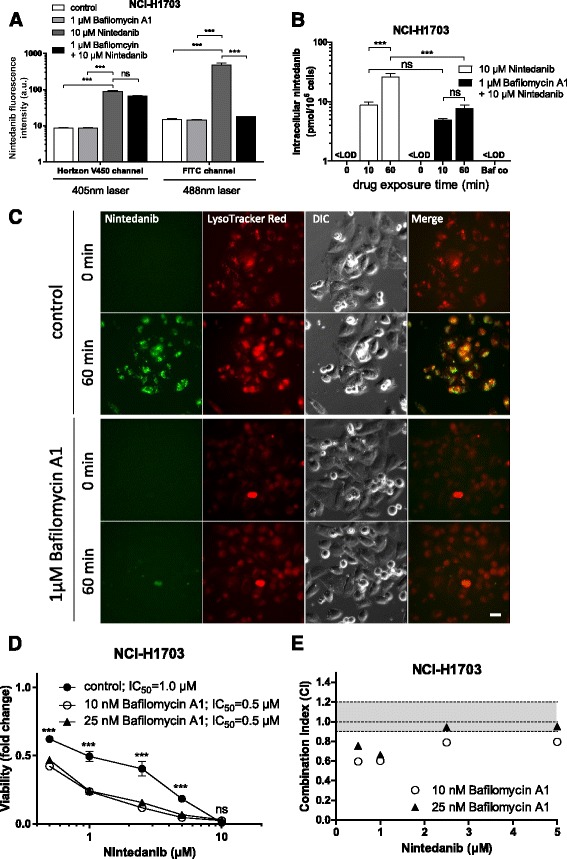
Lysosomal alkalization increases cytotoxic potential of nintedanib. **a** Impact of 1 h bafilomycin A1 pretreatment on the fluorescence activity of nintedanib in NCI-H1703 cells was measured after 1 h drug exposure by flow cytometry using the 405 nm and 488 nm lasers. ns, non-significant, *** *p* < 0.001, 2-way ANOVA, Tukey’s post-test. **b** Effect of 1 h bafilomycin A1 pretreatment on total intracellular nintedanib levels was determined at the indicated time-points by HPLC. <LOD, below limit of detection. *** *p* < 0.001, 2-way ANOVA, Tukey post-test. ns, non-significant. **c** Impact of 1 h bafilomycin A1 pretreatment on intralysosomal accumulation of 10 μM nintedanib was investigated at the indicated time-points by live cell microscopy. LysoTracker® Red served as marker for the lysosomal compartment. The scale bar indicates 10 μm. **d** Viability of NCI-H1703 lung cancer cells in the presence or absence of 10 and 25 nM bafilomycin A1 was analyzed by MTT assay after 72 h exposure to the indicated concentrations of nintedanib. *** *p* < 0.001, 2-way ANOVA, Tukey’s post-test. Asterisks indicate significance of difference at the respective nintedanib concentration points between control and both 10 nM and 25 nM bafilomycin A1. ns, non-significant; **e** Synergism of nintedanib/bafilomaycin A1 cotreatment of NCI-H1703 cells shown in (**d**) was determined calculating combination indices (CI) using CalcuSyn software. CI values below 0.9, between 0.9–1.1 or above 1.1 indicated synergism, additivity, and antagonism, respectively

The identified lysosomotropism of nintedanib prompted us to ask whether its cytotoxic potential might be enhanced upon disruption of the lysosomal integrity. Weakly basic lipophilic agents are commonly believed to become fully protonated in the acidic environment of lysosomes. This protonation renders them incapable of freely permeating lipid bilayers, thus trapping them in the lysosomal lumen [32]. To find out whether decreased lysosomal accumulation of nintedanib results in enhanced cytotoxicity, we exposed cells to the lysosomal vacuolar H^+^-ATPase (V-ATPase) inhibitor bafilomycin A1. This compound specifically blocks H^+^-ion influx and thus results in lysosomal alkalization [40]. Importantly, flow cytometric analysis as well as live cell microscopy revealed that bafilomycin A1 treatment completely abrogated green fluorescence of nintedanib in the investigated cell lines. Also for the blue nintedanib fluorescence, a moderate reduction was observed, albeit at a less pronounced level (1.3-fold in NCI-H1703, 1.7-fold in DMS114 and 1.5-fold in NCI-H520 cells), reaching significance only in DMS114 cells (Fig. 5a; Additional file 2: Figure S1A-C and Additional file 3: Table S2). Slightly reduced blue nintedanib fluorescence in the presence of Bafilomycin A1 was also detected by fluorescence microscopy (Additional file 2: Figure S1C). This indicated a trend towards reduced intracellular nintedanib levels in case of lysosomal alkalization. Direct quantification by HPLC indeed confirmed distinctly lower total intracellular nintedanib concentrations in the presence of bafilomycin A1 (1.8-fold and 3.4-fold after 10 min and 60 min, respectively) (Fig. 5b). In live cell microscopy, the blue nintedanib fluorescence exhibited a more diffuse intracellular distribution as compared to the green, exclusively intralysosomal signal (Additional file 2: Figure S1C). Furthermore, imaging of NCI-H1703 cells showed that lysosomal nintedanib accumulation was completely abolished in cells pretreated with bafilomycin A1 (Fig. 5c; Additional file 2: Figure S1C). This suggests that the observed green fluorescence activity originates from a specific state of nintedanib present exclusively in lysosomes with low pH. Interestingly, coincubation with non-toxic concentrations of bafilomycin A1 (Additional file 4: Figure S2A) led to a distinct sensitization of NCI-H1703 and NCI-H520 cells towards nintedanib (Fig. 5d, e and Additional file 4: Figure S2B, C, respectively). The slightly lower sensitization of NCI-H520 as compared to NCI-H1703 cells at both bafilomycin A1 concentrations (1.7-fold versus 2-fold sensitization, respectively) is likely to originate from the inherently lower sensitivity of the former cell line towards FGFR inhibition. Similarly, nintedanib co-incubation with subtoxic doses of chloroquine, another inhibitor of lysosomal acidification based in this case on proton capturing, also exerted synergistic effects, albeit to a slightly lesser extent (Additional file 4: Figure S2D-F). Together, this suggests that lysosomal alkalization suppresses protonation-based sequestration of nintedanib and, hence, loss of the respective green fluorescence emission. The lysosome exclusivity of the green nintedanib fluorescence likely arises from drug accumulation to such a high extent that results in compound precipitation. This is substantiated by the observation that crystalline nintedanib in cell-free conditions exhibited both green and blue fluorescence activity (Additional file 5: Figure S3). However, nintedanib dissolution resulted in complete and selective quenching of the green fluorescence, whereas the blue one was retained.

In conclusion, despite the observed decrease in overall intracellular nintedanib levels upon raising of the lysosomal pH, a concomitant increase of its cytosolic concentration leading to enhanced target interaction efficacy might be causative for the distinctly increased cytotoxic potential.

## Discussion

The lack or complexity of high resolution technologies and the need for labelled compound derivatives represents a major limitation on the study of intracellular distribution dynamics of pharmacological agents. The intrinsic, label-free and organelle-specific fluorescence activity of nintedanib presented in this study provides a powerful tool to dissect intracellular accumulation and distribution dynamics of this clinically approved small molecule TKI. The observation that lysosomal alkalization via V-ATPase inhibition sensitized lung cancer cells towards nintedanib suggests that protonation-based lysosomal sequestration represents a cell-intrinsic protection mechanism against this FGFR inhibitor. In accordance, various chemotherapeutic agents including doxorubicin, mitoxantrone and vincristine but also TKIs such as gefitinib, lapatinib and sunitinib have been reported to be subject to inactivating lysosomal sequestration [23, 30]. Together, these findings support a yet underestimated central role of lysosomal trapping in intrinsic resistance mechanisms against anticancer therapeutics including nintedanib.

A number of studies have previously reported that the physicochemical properties of small molecule inhibitors have an impact on their subcellular distribution [41]. Trapping of weakly basic pharmacological compounds through protonation in the lysosomal lumen, also termed ion-based sequestration, has been described to reduce the cytotoxic potential of numerous anticancer agents by scavenging them away from their respective sites of action [42]. Furthermore, it was shown that lysosomotropic anticancer drugs stimulate lysosomal biogenesis leading to further increased drug resistance [43]. A work by Trapp et al. reported a mathematical model to predict the lysosomotropism of pharmacological agents according to their physicochemical properties [26]. In this model, principal variables determining whether or not a given compound might accumulate in lysosomes are its lipophilicity, represented by the partition-coefficient (logP), and its tendency to become protonated at a given pH value, represented by the acid dissociation constant (pKa). Accordingly, Fu and colleagues predicted and confirmed the intralysosomal accumulation of the ABL1 inhibitors imatinib and nilotinib attributed to their weakly basic properties and pKa values greater than 8 by employing hyperspectral-stimulated Raman scattering imaging in BaF3 cells [21]. Based on its poorer water solubility, nilotinib but not imatinib concentrations rose to an extent high enough to even assume intralysosomal drug precipitation. With respect to the here-presented study, the piperazine moiety present in both nintedanib and imatinib can explain the comparable pKa values 7.9 and 8.1, respectively, which are ideal properties to observe lysosomotropism.

Interestingly, we detected different fluorescence characteristics associated with the intracellular nintedanib molecule. While short-wavelength blue fluorescence was detectable independent of lysosomal integrity and pH, the strong green fluorescence was strictly associated with nintedanib accumulated in acidic lysosomes. Hence, two intracellular conditions of this TKI can be distinguished by straight-forward fluorescence imaging or flow cytometry without any labelling. Moreover, the green fluorescence of nintedanib allowed precise quantification of the acidic compartment or lysosomal load under either live cell or fixed conditions (data not shown). Initially, we hypothesized that exclusively a protonated derivative of nintedanib might exert this specific fluorescence. Similar characteristics are utilized by acidotropic Lysosensor® probes to quantify dynamic changes in the acidic cellular compartment. However, in cell-free spectroscopic analyses modulation of various parameters such as pH shifts and addition of different protein species to closely mimic the lysosomal milieu did not result in any alterations in the fluorescence spectrum of nintedanib (data not shown). Furthermore, our HPLC data did not indicate any chemical metabolization of intracellular nintedanib accountable for a spectral shift. Alternatively, as in the case of nilotinib [21], nintedanib accumulation rates might be dramatic and result in intralysosomal concentrations exceeding drug solubility and thus resulting in aggregation or precipitation. This might also lead to altered fluorescence characteristics. Indeed, as we found crystalline nintedanib to exhibit –next to blue- also green fluorescence activity, drug precipitation is likely to be causative for the lysosome-specific green fluorescence. Independent which hypothesis holds true, the fluorescent derivative/state of nintedanib is extremely sensitive to loss of acidic pH as also short-term application of bafilomycin A1 switched off the respective signals in flow cytometric analyses already after a few minutes (data not shown). The underlying molecular processes are addressed in ongoing investigations. What concerns the slightly decreased overall intracellular nintedanib levels upon lysosomal de-acidification, we speculate that the high tropism for lysosomes promotes high intracellular nintedanib levels. Accordingly, lysosomes have high scavenging capacity that might even lead to nintedanib precipitation in the lysosomal lumen. Consequently, the overall lower intracellular nintedanib levels upon lysosomal de-acidification are likely to originate from decreased lysosomal sequestration- shifting the overall drug concentration equilibrium slightly towards the extracellular space. There the drug in its neutral state is expected to diffuse freely across the plasma membrane. At the same time, disrupting lysosomal sequestration may liberate higher drug amounts into the cytoplasm, resulting in more efficient FGFR inhibition.

The slightly lower potential of chloroquine as compared to bafilomycin A1 to sensitize cells against nintedanib may lie in their different modes of action. While bafilomycin A1 actively counteracts proton influx by H^+^−ATPase inhibition, chloroquine directly scavenges protons in the lysosomal lumen. We hypothesize that chloroquine is somewhat less potent in decreasing lysosomal nintedanib levels, as nintedanib is also a strongly lysosomotropic compound, itself scavenging protons from chloroquine in a competitive manner.

Preliminary analyses of cryosections derived from allograft tumors of orally treated mice suggest that this method might even be applicable for pharmacokinetic studies of organismal and subcellular nintedanib distribution in vivo.

Our findings might also be clinically relevant. For instance, key characteristics of the lysosomal compartment or related processes in tumors, such as lysosomal load or autophagic activity, might have crucial impacts on nintedanib response. Furthermore, prevention of subcellular trapping via lysosome-alkalization represents a feasible strategy to increase drug availability at the target site and, consequently, to circumvent cell-intrinsic nintedanib resistance.

## Conclusion

We have uncovered that the multikinase inhibitor nintedanib exhibits intrinsic organelle-specific fluorescence properties which can be exploited to detect this agent at subcellular levels in cell culture as well as in allografted tumor tissue of orally-treated mice. The observed lysosomal sequestration of this compound is likely to constitute a cell-intrinsic resistance mechanism, lowering cytosolic drug concentrations and, thus, the FGFR inhibition-based cytotoxic potential. Our findings suggest that key characteristics of lysosomal compartments in tumors, such as lysosome number or size, might have an impact on sensitivity towards nintedanib. Additionally, combination approaches with agents targeting lysosomal pH - including clinically used remedies like chloroquine - should represent an innovative approach to circumvent intrinsic nintedanib resistance but might also distinctly alter the pharmacokinetic properties of this multikinase inhibitor.

## Additional files

Additional file 1: Table S1. Fluorescence properties of intracellular nintedanib. (DOCX 15 kb)

Additional file 2: Figure S1. Lysosomal alkalization selectively abrogates green fluorescence activity of nintedanib in DMS114 and NCI-H520 cells. (PPTX 1566 kb)

Additional file 3: Table S2. Impact of lysosomal alkalization by bafilomycin A1 on nintedanib fluorescence. (DOCX 14 kb)

Additional file 4: Figure S2. Lysosomal alkalization leads to sensitization towards nintedanib. (PPTX 335 kb)

Additional file 5: Figure S3. Crystalline nintedanib exhibits both blue and green fluorescence properties. Cell-free nintedanib fluorescence properties in crystalline form or dissolved in DMSO were analyzed by fluorescence microscopy using DIC, FITC and DAPI channels. (PPTX 718 kb)

## Abbreviations

ABCB1: ATP-binding cassette transporter B1
aCGH: Array comparative genomic hybridization
CI: Combination index
DIC: Differential interference contrast
EGFR: Epidermal growth factor receptor
FGFR: Fibroblast growth factor receptor
MCC: Manders’ Colocalization Coefficient
MTT: (3-(4,5-Dimethylthiazol-2-yl)-2,5-Diphenyltetrazolium Bromide)
NSCLC: Non-small cell lung cancer
PDGFR: Platelet-derived growth factor receptor
PFA: Paraformaldehyde
RTK: Receptor tyrosine kinase
SCLC: Small cell lung cancer
SD: Standard deviation
TKI: Tyrosine kinase inhibitor
VEGFR: Vascular endothelial growth factor receptor
