# Supplementary material for: Intrinsic fluorescence of the clinically approved multikinase inhibitor nintedanib reveals lysosomal sequestration as resistance mechanism in FGFR-driven lung cancer
Source: J Exp Clin Cancer Res. 2017 Sep 7;36:122. doi: 10.1186/s13046-017-0592-3 (PMC5590147; doi:10.1186/s13046-017-0592-3)
Supplement: Supplementary file 1 — Fluorescence properties of intracellular nintedanib. (DOCX 15 kb) [file 13046_2017_592_MOESM1_ESM.docx]

| Table S1. Fluorescence properties of intracellular nintedanib. | | | | | |
| --- | --- | --- | --- | --- | --- |
| Cell line/  laser (nm) | Channel | bandpass filter  (nm) | mean fluorescence intensity  (a.u. mean ± SD)^a^ | | relative fluorescence increase^b^ |
|  |  |  | control | Nintedanib |  |
| NCI-H1703 |  |  |  |  |  |
| 405 | Horizon V450 | 450/40 | 7.99 ± 0.08 | 166.15 ± 9.33 | 20.8^***^ |
| 488 | FITC | 530/30 | 10.67 ± 0.24 | 992.00 ± 84.75 | 93.0^***^ |
| DMS114 |  |  |  |  |  |
| 405 | Horizon V450 | 450/40 | 17.47 ± 0.32 | 28.65 ± 0.68 | 1.6^***^ |
| 488 | FITC | 530/30 | 13.65 ± 0.28 | 1,134.26 ± 50.08 | 83.1^***^ |
| NCI-H520 |  |  |  |  |  |
| 405 | Horizon V450 | 450/40 | 11.34 ± 0.10 | 318.18 ± 7.19 | 28.1^***^ |
| 488 | FITC | 530/30 | 13.25 ± 0.31 | 1,857.35 ± 82.08 | 140.2^***^ |
| ^a^ Cells were treated for 1 hour with 10 µM nintedanib and analyzed by flow cytometry. a.u., arbitrary units.  ^b^ Increase in fluorescence intensity of nintedanib-treated cells is given relative to untreated controls. | | | | | |

^***^ p<0.001, 2-way ANOVA, Bonferroni post-test.
