# Supplementary material for: Intrinsic fluorescence of the clinically approved multikinase inhibitor nintedanib reveals lysosomal sequestration as resistance mechanism in FGFR-driven lung cancer
Source: J Exp Clin Cancer Res. 2017 Sep 7;36:122. doi: 10.1186/s13046-017-0592-3 (PMC5590147; doi:10.1186/s13046-017-0592-3)
Supplement: Supplementary file 2 — Lysosomal alkalization selectively abrogates green fluorescence activity of nintedanib in DMS114 and NCI-H520 cells. (PPTX 1566 kb) [file 13046_2017_592_MOESM2_ESM.pptx]

## Slide 1
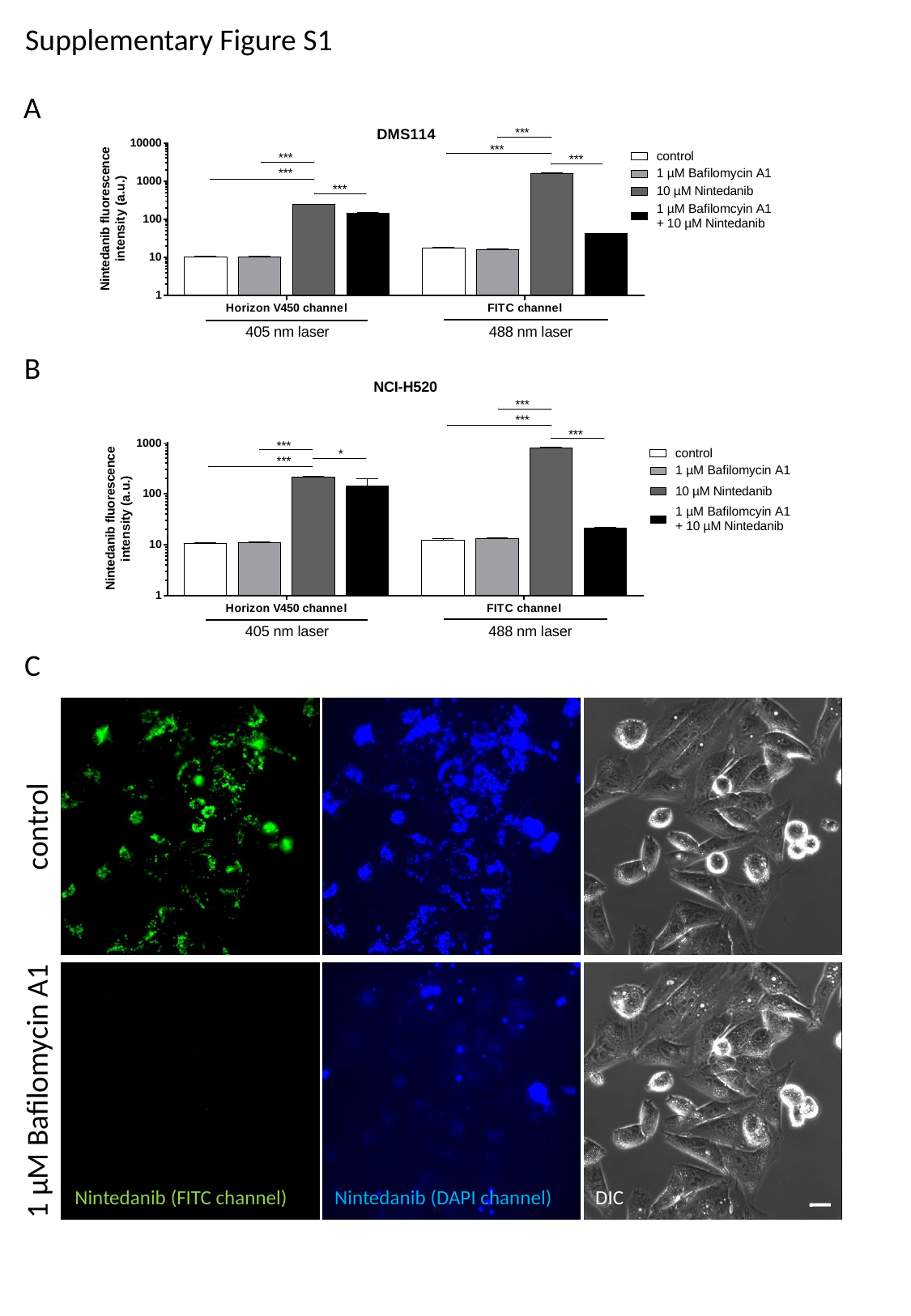

Supplementary Figure S1
A
B
C
control
1 µM Bafilomycin A1
Nintedanib (FITC channel)
Nintedanib (DAPI channel)
DIC
