# Supplementary material for: Intrinsic fluorescence of the clinically approved multikinase inhibitor nintedanib reveals lysosomal sequestration as resistance mechanism in FGFR-driven lung cancer
Source: J Exp Clin Cancer Res. 2017 Sep 7;36:122. doi: 10.1186/s13046-017-0592-3 (PMC5590147; doi:10.1186/s13046-017-0592-3)
Supplement: Supplementary file 3 — Impact of lysosomal alkalization by bafilomycin A1 on nintedanib fluorescence. (DOCX 14 kb) [file 13046_2017_592_MOESM3_ESM.docx]

| Table S2. Impact of lysosomal alkalization by bafilomycin A1 on nintedanib fluorescence. | | | | | | | |
| --- | --- | --- | --- | --- | --- | --- | --- |
| Cell line/  laser (nm) | Channel | bandpass filter  (nm) | mean fluorescence intensity (a.u., mean ± SD)^a^ | | | | relative fluorescence decrease^b^ |
|  |  |  | control | Bafilomycin A1 | Nintedanib | Nintedanib + Bafilomycin A1 |  |
| NCI-H1703 | |  |  |  |  |  |  |
| 405 | Horizon V450 | 450/40 | 8.90  ± 0.08 | 8.74  ± 0.08 | 89.32  ± 4.77 | 68.34  ± 0.35 | 1.3^ns^ |
| 488 | FITC | 530/30 | 15.07  ± 0.51 | 14.38  ± 0.26 | 495.35  ± 43.14 | 18.04  ± 0.20 | 27.5^***^ |
| DMS114 | |  |  |  |  |  |  |
| 405 | Horizon V450 | 450/40 | 10.55  ± 0.29 | 10.68  ± 0.20 | 249.54  ± 1.29 | 147.22  ± 1.32 | 1.7^***^ |
| 488 | FITC | 530/30 | 17.91  ± 0.21 | 16.83  ± 0.39 | 1,641.73  ± 39.96 | 42.87  ± 0.69 | 38.3^***^ |
| NCI-H520 | |  |  |  |  |  |  |
| 405 | Horizon V450 | 450/40 | 10.78  ± 0.29 | 11.27  ± 0.12 | 221.36  ± 4.13 | 146.11  ± 56.34 | 1.5 ^ns^ |
| 488 | FITC | 530/30 | 12.62  ± 0.35 | 13.44  ± 0.26 | 813.62  ± 36.20 | 21.39  ± 0.29 | 38.0^***^ |
| ^a^ Cells were pretreated with bafilomycin A1 for 1 hour, incubated with 10 µM nintedanib for 1 hour and measured by flow cytometry. a.u., arbitrary units.  ^b^ Relative nintedanib fluorescence decrease by coincubation with bafilomycin A1 (nintedanib / (nintedanib + bafilomycin A1)). ^ns^ non significant; ^***^  p<0.001, 2-way ANOVA, Bonferroni post-test. | | | | | | | |
