# Supplementary figures and images for: Intrinsic fluorescence of the clinically approved multikinase inhibitor nintedanib reveals lysosomal sequestration as resistance mechanism in FGFR-driven lung cancer
Source: J Exp Clin Cancer Res. 2017 Sep 7;36:122. doi: 10.1186/s13046-017-0592-3 (PMC5590147; doi:10.1186/s13046-017-0592-3)

## Slide 1
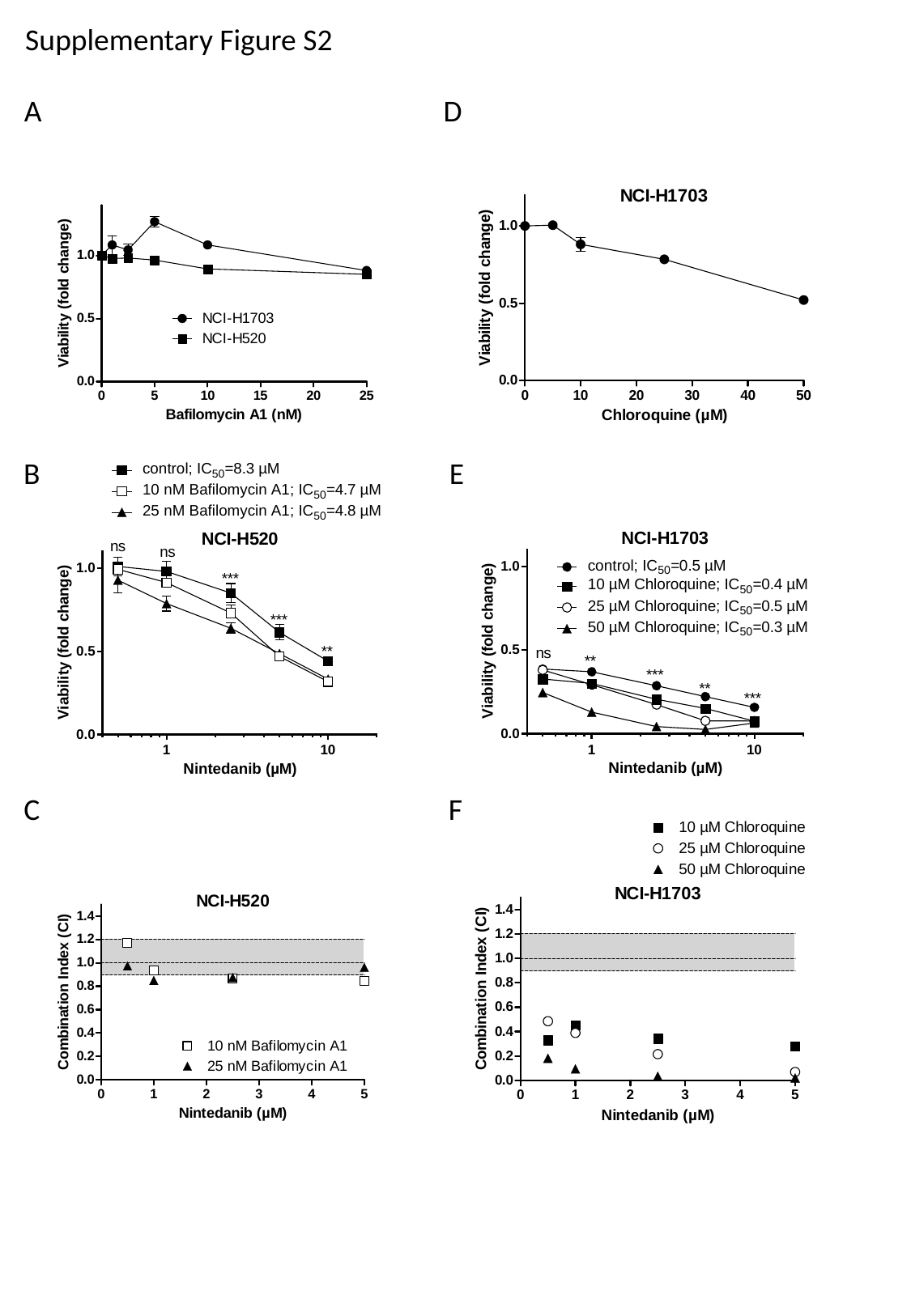

Supplementary Figure S2
A
D
B
E
C
F

Supplement: Supplementary file 4 — Lysosomal alkalization leads to sensitization towards nintedanib. (PPTX 335 kb) [file 13046_2017_592_MOESM4_ESM.pptx]
