# Supplementary material for: Intrinsic fluorescence of the clinically approved multikinase inhibitor nintedanib reveals lysosomal sequestration as resistance mechanism in FGFR-driven lung cancer
Source: J Exp Clin Cancer Res. 2017 Sep 7;36:122. doi: 10.1186/s13046-017-0592-3 (PMC5590147; doi:10.1186/s13046-017-0592-3)
Supplement: Supplementary file 5 — Crystalline nintedanib exhibits both blue and green fluorescence properties. Cell-free nintedanib fluorescence properties in crystalline form or dissolved in DMSO were analyzed by fluorescence microscopy using DIC, FITC and DAPI channels. (PPTX 718 kb) [file 13046_2017_592_MOESM5_ESM.pptx]

## Slide 1
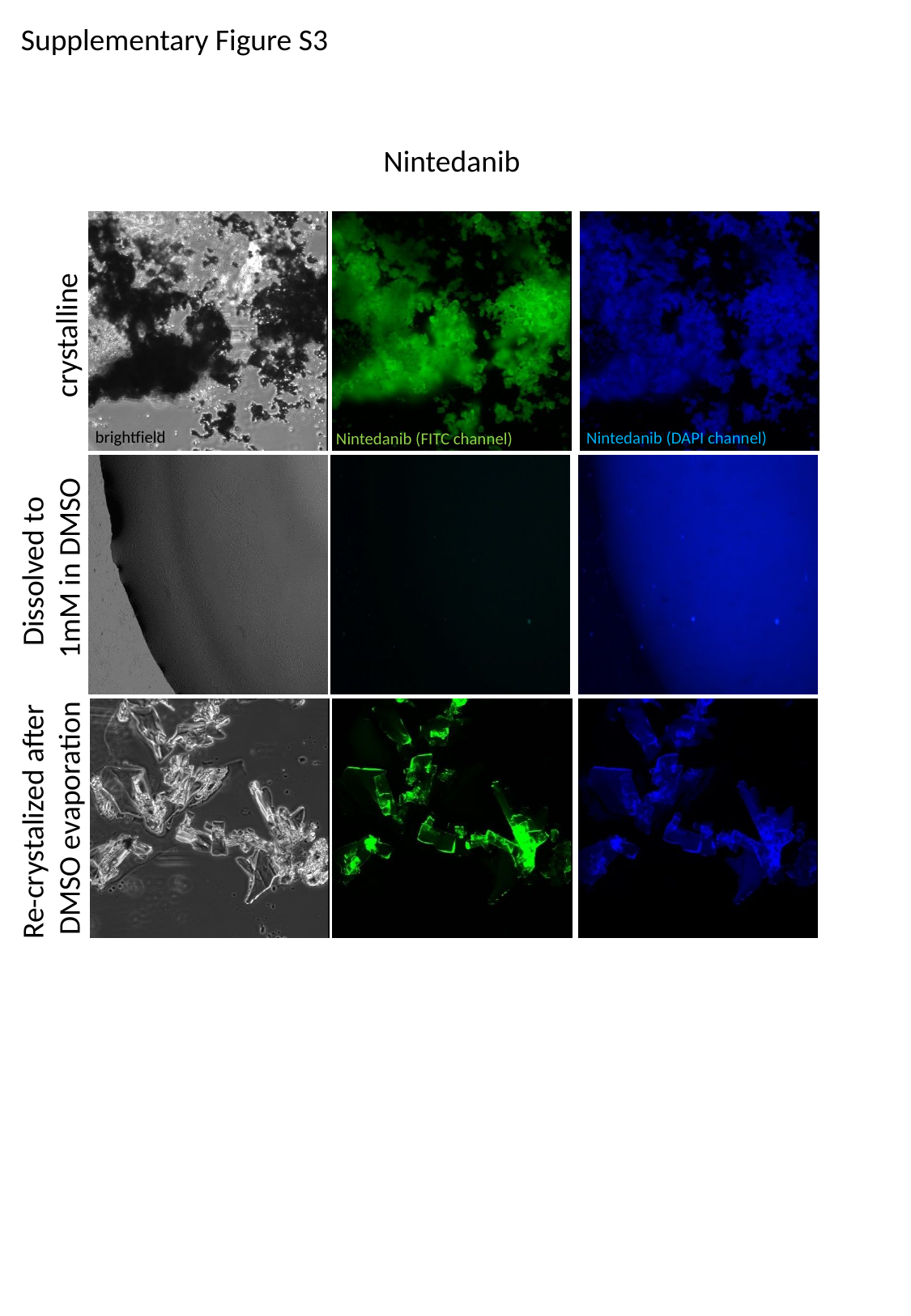

Supplementary Figure S3
Nintedanib
crystalline
brightfield
Nintedanib (DAPI channel)
Nintedanib (FITC channel)
Dissolved to
1mM in DMSO
Re-crystalized after
DMSO evaporation
